# Impact of blood lipid levels on breast cancer prognosis: a systematic review and meta-analysis

**DOI:** 10.3389/fonc.2025.1496468

**Published:** 2025-07-01

**Authors:** Jiaqing Song, Ying Jin, Qinghong Yu, Hongting Wu, Xiufei Gao

**Affiliations:** The First Affiliated Hospital of Zhejiang Chinese Medical University, Hangzhou, Zhejiang, China

**Keywords:** blood lipid levels, breast cancer, prognosis, disease-free survival, overall survival

## Abstract

**Background:**

Breast cancer has emerged as the predominant malignant neoplasm globally, with potential implications for patient prognosis based on blood lipid profiles. This study aims to systematically review and meta-analyze the influence of lipid levels on the prognostic outcomes of individuals with breast cancer.

**Methods:**

A thorough search was performed across multiple academic databases, including Embase, Cochrane, PubMed, Web of Science, CNKI, and Wanfang Database, up to March 2024. A meta-analysis was conducted to assess the impact of total cholesterol (TC), triglycerides (TG), low-density lipoprotein-cholesterol (LDL-C), and high-density lipoprotein-cholesterol (HDL-C) on the prognosis of Breast Cancer. The primary outcome measure was hazard ratios (HR) for overall survival (OS) and/or disease-free survival (DFS).

**Results:**

Eight studies meeting inclusion criteria from a total of 13,292 were included in the meta-analysis. The systematic review and meta-analysis demonstrate an association between lower HDL-C levels and poorer survival outcomes. However, the statistical analysis did not find significant associations between HDL-C, TG, and LDL-C levels and the prognosis of breast cancer patients.

**Conclusion:**

While our analysis reveals a link between reduced HDL-C levels and unfavorable survival outcomes, the statistical evidence does not support significant connections between HDL-C, TG, and LDL-C concentrations and the prognostic landscape for breast cancer patients. Further research is warranted to explore these relationships more comprehensively.

**Systematic Review Registration:**

https://www.crd.york.ac.uk/PROSPERO, identifier CRD42021297118.

## 1 Introduction

Based on the most recent data published by the International Agency for Research on Cancer, the global incidence of new cases of breast cancer in 2022 has reached 2.3 million, making it the second most prevalent form of cancer worldwide. Furthermore, breast cancer has now surpassed gastric cancer to become the fourth leading cause of cancer-related mortality globally (2024). In China, the incidence of female breast cancer reached 357,200 cases in 2022, positioning it as the fifth most prevalent cancer (1) and supplanting colon and rectal cancer as the fifth leading cause of cancer mortality among women (2). Factors such as patient lifestyle, adjuvant therapy for breast cancer, and the significant reduction in estrogen levels due to menopause can contribute to the development of dyslipidemia in individuals with breast cancer (3). Nature has reported that obese individuals are at a heightened risk for developing breast cancer (4, 5), with a 25% increase in mortality among breast cancer patients who are obese (6). Additionally, a meta-analysis has indicated that obesity is linked to poorer disease-free survival (DFS) and overall survival (OS) across all subtypes of breast cancer (7). The prevalence of obesity among breast cancer patients is as high as 70% (8), and obese individuals typically exhibit elevated blood lipid levels (9). Following chemotherapy and endocrine therapy, blood lipid levels tend to remain abnormal in this population (10, 11). Hence, blood lipids play a role in influencing the prognosis of breast cancer, yet there is a scarcity of systematic reviews examining the relationship between lipids and breast cancer prognosis.

The findings of a meta-analysis examining the relationship between metabolic syndrome and breast cancer prognosis revealed conflicting results regarding the impact of dyslipidemia on breast cancer outcomes (12). Given the lack of a clear correlation, further research is warranted to explore the potential role of lipids in breast cancer prognosis. Importantly, identifying factors that influence the participation and adherence to breast cancer screening programs, such as the finding by Esra Bayrakceken et al. (13) that high blood lipid level affected the time women get mammography screening, can provide valuable insights into improving early detection and treatment outcomes. Similarly, understanding the relationship between lipid profiles and breast cancer prognosis may offer critical information for developing targeted interventions to enhance patient outcomes and reduce mortality rates. Thus, this study aims to fill this gap in the literature by systematically reviewing the available evidence on the association between blood lipid levels and breast cancer prognosis.

## 2 Methods

### 2.1 Protocol and registration

This systematic review has been conducted following the guidelines outlined in the Preferred Reporting Items for Systematic Reviews and Meta-Analyses (PRISMA) (14) and has been registered in the PROSPERO International Register of Systematic Reviews under the registration number CRD42021297118.

### 2.2 Search strategy

A systematic search of electronic databases, including Embase, Cochrane, PubMed, Web of Science, CNKI, and Wanfang Database, was conducted by trained librarians to identify studies on the relationship between “breast cancer” and lipid profiles (HDL-C, LDL-C, Total cholesterol, Triglycerides) from inception to March 2024. References from included studies and review articles will be examined to locate additional relevant studies. The search strategy may be updated before final data analysis to include any newly published studies. The full search strategy is provided in online **Supplementary Box 1**.

### 2.3 Inclusion/exclusion criteria

Inclusion criteria encompassed clinical trials and cohort studies without type restrictions, involving participants over 18 years old diagnosed with primary breast cancer. Outcome indicators included hazard ratios for overall survival and/or disease-free survival in breast cancer patients with varying lipid levels. Exclusion criteria comprised non-clinical research such as cell studies and animal experiments, as well as redundant publications and literature reviews. Additionally, patients with other malignancies were excluded from clinical trials.

### 2.4 Data management and analysis

The results obtained from the electronic database search were imported into EndNote X9, a software utilized for managing academic literature, where duplicate titles were eliminated. Titles and abstracts were reviewed by two authors, with any discrepancies resolved through discussion. Articles deemed potentially eligible were subjected to full-text screening. A minimum of two reviewers independently assessed the full-text articles, and data extraction was conducted by two authors using standardized forms. Study authors were contacted for supplementary information as needed.

The meta-analysis examined the association between lipid levels and prognosis in breast cancer patients. Heterogeneity among the included studies was assessed using the *I^2^* statistic, which quantifies the proportion of total variance attributable to differences between studies rather than random error. The analyses were performed using Review Manager 5.

### 2.5 Assessment of study quality

Two authors conducted a risk of bias assessment utilizing the Newcastle-Ottawa scale (15). This assessment was applied to both observational and interventional studies due to the authors’ *post hoc* analyses in clinical trials, where populations were randomized to different treatments. In these trials, levels of HDL-C, LDL-C, TC, and TG were found to be unrelated to the randomization process.

## 3 Results

### 3.1 Study selection

**Figure 1:**
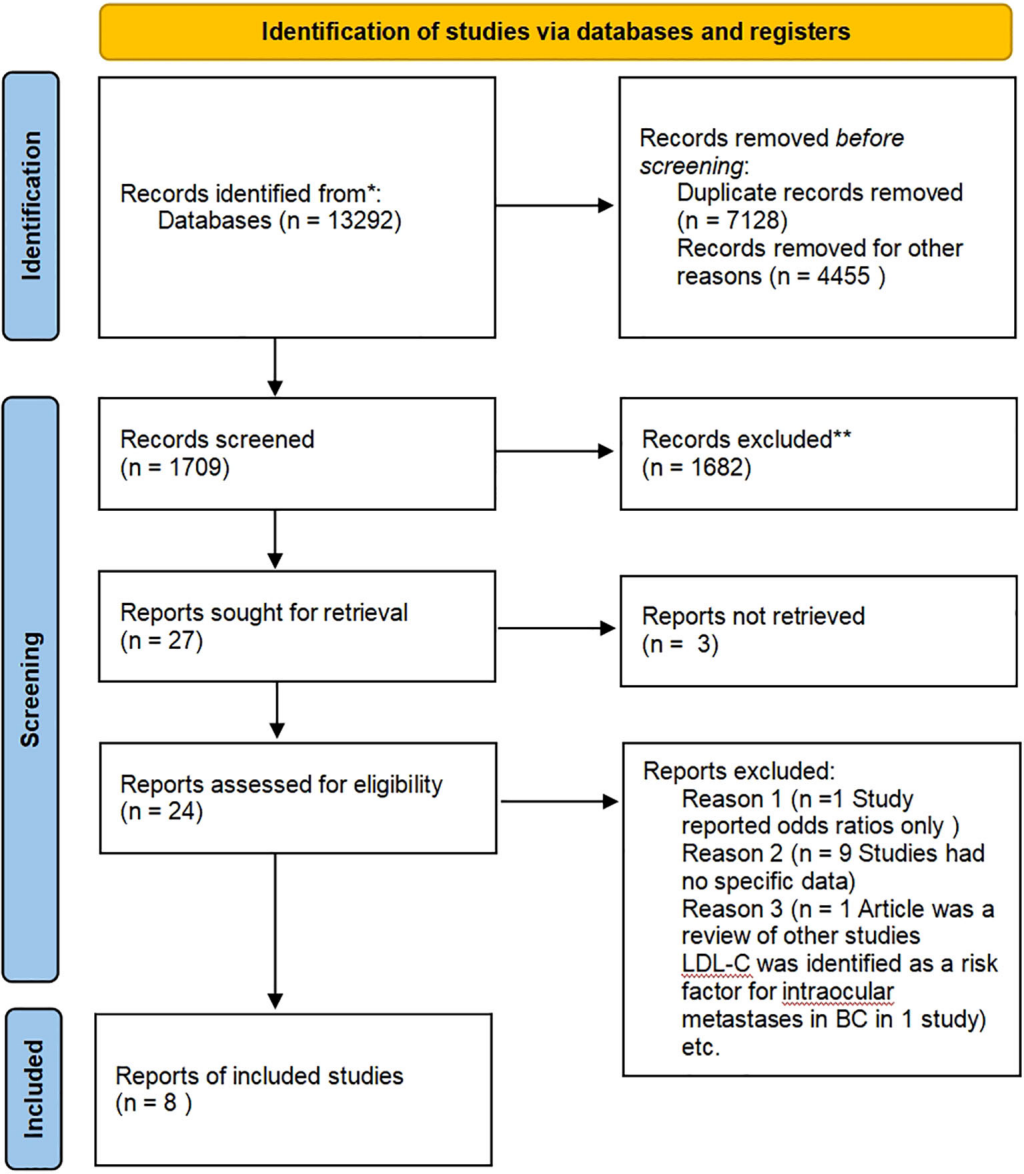
PRISMA diagram outlining the search strategy and selection of studies included in this review.

**Figure 1** presents a summary of the publications reviewed. A literature search of electronic databases yielded 13,292 results, with secondary searches of the reference lists of retained full-text articles yielding no additional articles. After removing duplicates, a total of 6,164 studies were included, with 542 focusing on HDL-C, 1,268 on LDL-C, 1,798 on TG, and 2,559 on TC (some studies focused on multiple lipid indicators simultaneously). The majority of studies were excluded based on titles and abstracts. Following the application of pre-screening eligibility criteria, 27 full-text articles were evaluated. Three full texts were unavailable for retrieval. Sixteen articles were excluded with justification, while eight articles met the inclusion criteria.

### 3.2 Study characteristics

The attributes of the studies incorporated are delineated in **Table 1**, encompassing a total of 7186 participants. All eight studies were cohort studies focusing on female breast cancer patients, with sample sizes ranging from 202 to 3499. The predominant geographical location of the studies was China.

**Table 1:** Summary of basic characteristics of selected studies for meta-analysis.

Author	Year	Country	Sample size	Median Age (years)	Follow-up (months)
Li X (16)	2017	China	1044	47 (22-85)	144.6
Fan Y (17)	2015	China	1391	49 (22-91)	145
Rodrigues Dos Santos C (18)	2014	Portugal	244	58 (29-91)	25
Dai D (19)	2016	China	221	45 (25-79)	142
Cheng-wei L (20)	2022	China	341	47 (21-79)	100
Xiaorong L (21)	2015	China	244	52 (29-85)	92
Wenxia W (22)	2021	China	202	48 (37-59)	36
Dong S (10)	2023	China	3499	NA	60.4

### 3.3 Quality appraisal results

Given that the studies incorporated in the analysis are all cohort studies, the Newcastle-Ottawa Quality Assessment Scale for cohort studies was employed for the purpose of evaluation. The quality assessment outcomes of the included studies are presented in **Table 2**.

**Table 2:** The Newcastle-Ottawa quality assessment scale.

Study, year	Selection				Comparability	Outcome			Overall Score
	Exposed representation	Nonexposed selection	Determination of criteria for dyslipidemia	Outcome absent at study start	Adjustment by age and nodal status or stage	Outcome assessment	Follow-up length	Adequacy of follow-up	
16	Y	Y	Y	Y	Y	Y	Y	Y	9
17	Y	Y	Y	Y	Y	Y	Y	Y	9
18	Y	Y	Y	Y	Y	Y	Y	Y	9
19	Y	Y	Y	Y	Y	Y	Y	Y	9
20	Y	Y	Y	Y	Y	Y	Y	Y	9
21	Y	Y	Y	Y	Y	Y	Y	Y	9
22	Y	Y	Y	Y	Y	Y	Y	Y	9
10	Y	Y	Y	Y	Y	Y	Y	Y	9

Y, Yes.

### 3.4 Key findings of the included studies

Five studies were conducted to examine the impact of HDL-C levels on breast cancer prognosis. One retrospective study by Li X et al. (16) demonstrated a significant association between lower HDL-C levels and poorer OS in multivariate analysis (HR=0.528; 95%CI: 0.302-0.923; *P*=0.025), although no statistical significance was found for DFS. Another retrospective study by Fan Y et al. (17) found that low HDL-C levels were independently associated with poor prognosis for recurrence-free survival (RFS) (HR=3.266, 95%CI:2.087-5.112, *P*<0.0001) and OS (HR=3.071, 95%CI:1.732-5.445, *P*<0.0001) in patients with triple-negative breast cancer (TNBC). Decreased levels of HDL-C have been associated with poorer outcomes in terms of RFS and OS specifically in patients with TNBC, as opposed to non-TNBC patients. LI C et al. (20) demonstrated that elevated HDL-C levels were correlated with improved DFS in TNBC patients in a COX multivariate analysis (HR=3.916, 95% CI:1.355-11.313, *P*=0.012). Additionally, a study by Wu W et al. (22) revealed that a baseline HDL-C level below 1.14 mmol/L was associated with increased risk of recurrence (HR=2.907, 95% CI:1.024-8.255, *P*=0.045) and mortality (HR=8.718, 95% CI: 1.148-66.198, *P*=0.036) after adjusting for factors such as menopausal status, BMI, blood pressure, and blood glucose levels. A study by Dong S et al. (10) found that HDL was related to more favorable clinical outcomes (HR=0.70, 95% CI: 0.56–0.89, *P*<0.01). No significant relationship with OS was observed.

**Figure 2:**
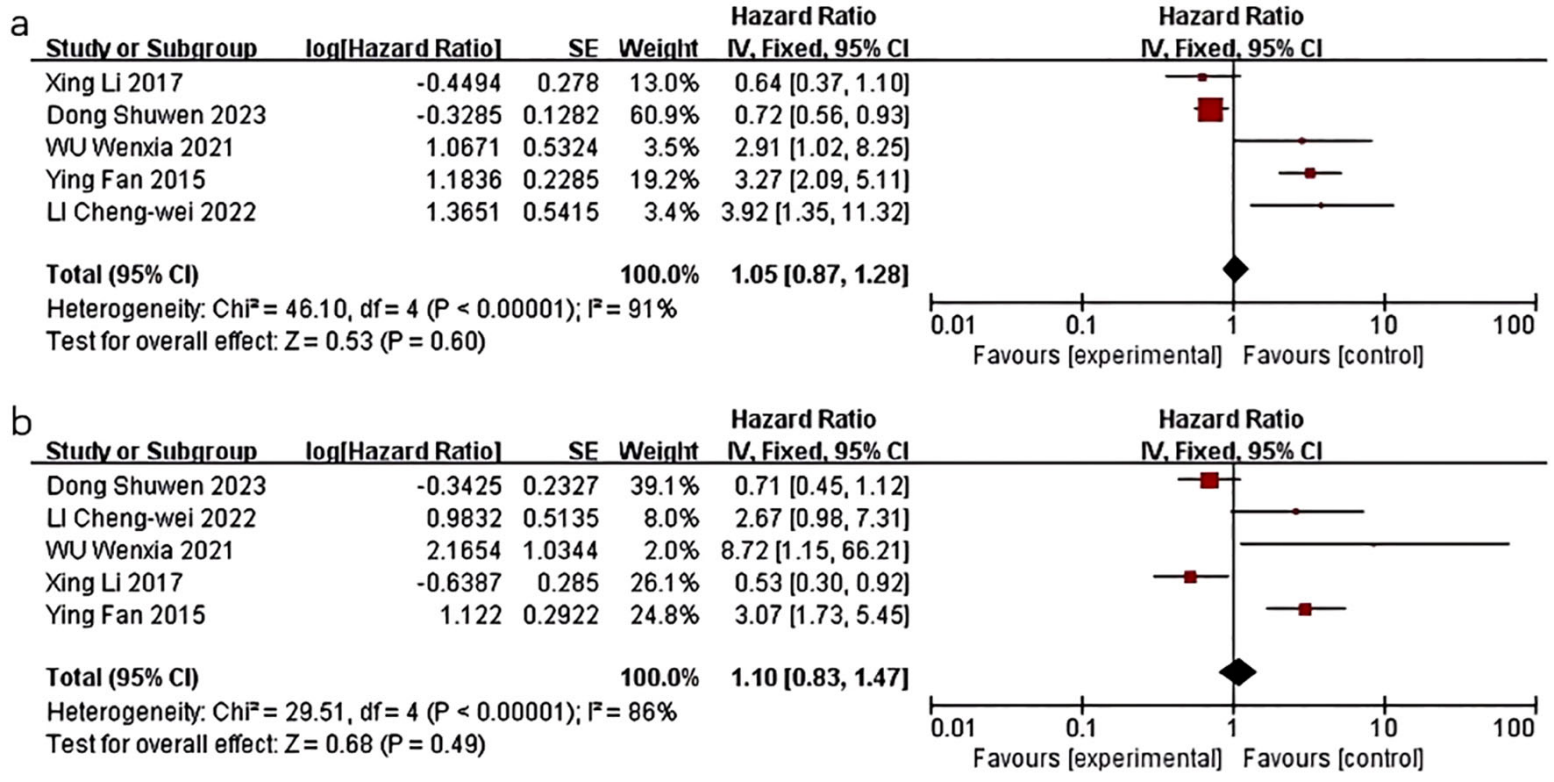
Association of lower HDL-C levels with DFS and OS of breast cancer patients. **(a)** Association of lower HDL-C levels with DFS of breast cancer patients; **(b)** Association of lower HDL-C levels with OS of breast cancer patients.

Five studies were included in the quality-effect meta-analysis (**Figure 2**), revealing an association between decreased levels of HDL-C and diminished disease-free survival (HR=1.05, 95% CI:0.87-1.28, *P*=0.60) and overall survival (HR=1.10, 95% CI:0.83-1.47, *P*=0.49) in breast cancer patients. In summary, lower HDL-C levels are correlated with unfavorable prognostic outcomes in breast cancer patients, characterized by reduced survival duration, heightened likelihood of disease recurrence, metastasis, and mortality, but there was no statistical significance.

Five studies examined the correlation between LDL-C levels and breast cancer prognosis. Rodrigues dos Santos et al. (18) discovered that LDL-C levels exceeding 117 mg/dL were linked to decreased disease-free survival (HR=0.129; 95% CI:0.017-0.978; *P*=0.048). LI C et al. (20) also observed that lower LDL-C levels were associated with improved disease-free survival (HR=0.412, 95% CI: 0.180-0.946, *P*=0.037). In the study conducted by Lin Xiaorong et al. (21), the Cox regression model demonstrated a significantly poorer DFS in the group with LDL-C levels exceeding 3.08 mmol/L compared to those with LDL-C levels at or below 3.08 mmol/L. This finding suggests that LDL-C levels serve as independent prognostic factors for DFS (HR=0.4848; 95% CI: 0.2556-0.9194; *P*=0.0026). Conversely, in the research conducted by Li, X et al. (16)and Dong S et al. (10), no statistically significant association was observed between LDL-C levels and either DFS or OS.

**Figure 3:**
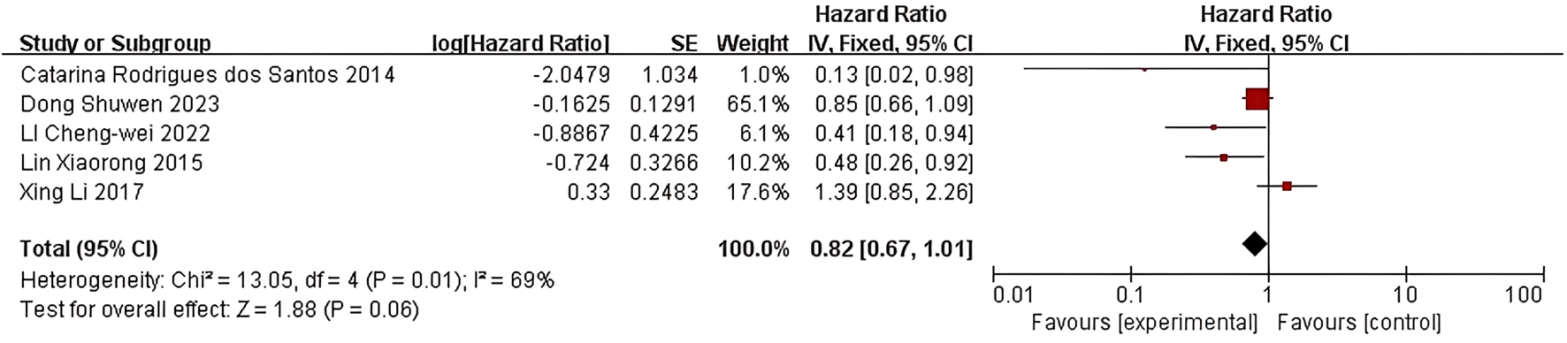
Association of lower LDL-C levels with DFS of breast cancer patients.

Five studies were included in the quality-effect meta-analysis (**Figure 3**), which indicated no significant association between lower LDL-C levels and disease-free survival (HR=0.82, 95% CI:0.67-1.01, *P*=0.06) in breast cancer patients.

In the context of TG levels and the prognosis of breast cancer, Li et al. (16) discovered that decreased TG levels were significantly linked to a poorer DFS outcome (HR=0.569; 95%CI: 0.370-0.873; *P*=0.010). Conversely, LI C et al. (20) found that low TG levels were associated with a beneficial DFS outcome (HR=0.408, 95% CI: 0.175-0.951, *P*=0.038). Similarly, Danian Dai et al. (19) observed that in univariate analysis, shorter DFS was correlated with higher TG levels (HR=1.811; 95%CI:1.174-2.793; *P*=0.007), as well as shorter OS (HR=1.710, 95%CI:1.099-2.661, *P*=0.017). However, the results of the multivariate survival analysis model indicated that TG levels were not statistically significant in relation to DFS and OS outcomes. Dong S et al. (10) found that patients with sustained high levels of TG had poorer RFS (HR=1.90, 95% CI:1.16–3.11), but no statistically significant association was observed between TG levels and OS.

**Figure 4:**
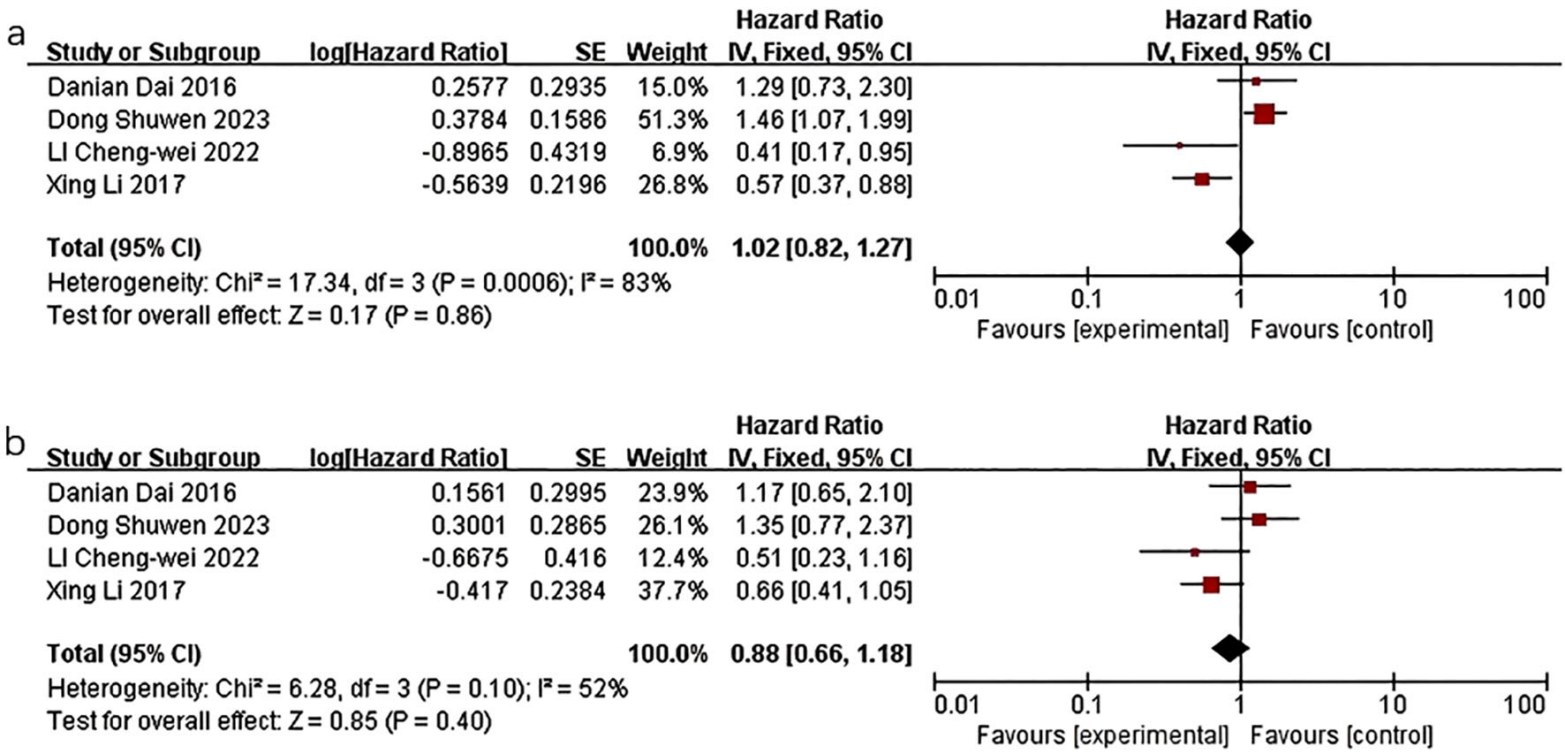
Association of lower TG levels with DFS and OS of breast cancer patients. **(a)** Association of lower TG levels with DFS of breast cancer patients. **(b)** Association of lower TG levels with OS of breast cancer patients.

The quality-effect meta-analysis incorporated four studies and indicated that there was no statistically significant correlation between triglyceride levels and disease-free survival (HR=1.02, 95%CI 0.82-1.27, *P*=0.86) or overall survival (HR=0.88, 95%CI:0.66-1.18, *P*=0.40) (**Figure 4**).

The study conducted by Lin Xiaorong et al. (21) utilized a Cox regression model to demonstrate a lower DFS rate in the group with TC levels greater than or equal to 5.2 mmol/L compared to those with TC levels less than 5.2 mmol/L. This finding indicates that TC is an independent prognostic factor for DFS, with a hazard ratio of 0.4747 (95%CI: 0.2732-0.8245; *P*=0.008). However, study conducted by Dong S et al. (10) observed that no significant association was observed between TC levels and prognosis.

## 4 Discussion

This systematic review and meta-analysis demonstrate an association between lower HDL-C levels and poorer survival outcomes. However, the statistical analysis did not find significant associations between HDL-C, TG, and LDL-C levels and the prognosis of breast cancer patients.

This study undertook a systematic review of clinical literature examining the impact of lipid levels on breast cancer prognosis. The review revealed various shortcomings in the existing studies, such as inconsistent criteria for evaluating dyslipidemia and variations in the demographics of the study populations. These variations stem from differences in factors such as race, age, breast cancer subtype, geographic location, and dietary patterns, potentially resulting in disparate prognostic outcomes across studies.

In the United States, the prevalence of obesity is 41.9% (23), with individuals of African descent exhibiting the highest susceptibility and those of Asian descent displaying the lowest susceptibility (24). Conversely, in China, the prevalence of overweight/obesity stands at 48.9%, with a greater representation of males (25). It is widely recognized that obesity serves as a risk factor for the initiation and advancement of breast cancer, and dyslipidemia, a prevalent complication of obesity (25), and it must not be overlooked for its potential impact on breast cancer. The variations in blood lipid levels among breast cancer patients of diverse racial and geographical backgrounds necessitate further investigation into their respective impacts on the prognosis of the disease.

Moreover, research indicates that there are varying correlations between lipid levels and distinct subtypes of breast cancer. Specifically, studies have demonstrated that estrogen receptor (ER) and progesterone receptor (PR) positive breast cancer exhibit a negative association with HDL-C and LDL-C levels, with HDL-C levels being independently linked to ER and PR positive breast cancer in postmenopausal women (26). Furthermore, abnormal levels of TC, LDL-C, HDL-C, and apolipoprotein A1 (ApoA1) are closely associated with different molecular subclasses of breast cancer and are correlated with the expression of the Ki-67 protein. Notably, abnormalities in TC, LDL-C, HDL-C, and ApoA1 are more prevalent in the TNBC and human epidermal growth factor receptor 2 (HER2)-positive breast cancer subtypes compared to the luminal subtype (27). The assessment of lipid levels in individuals diagnosed with primary invasive breast cancer enables the identification of potential diagnostic indicators and therapeutic targets for the disease, offering novel insights that may enhance prognostic accuracy and inform tailored treatment strategies for distinct molecular subtypes of breast cancer.

Dyslipidemia is a significant risk factor for cardiovascular disease (28), with elevated LDL-C levels being particularly linked to an increased risk of atherosclerotic cardiovascular disease resulting from the accumulation of plaque within arterial walls (29). There exists compelling evidence supporting the notion that the trajectory of atherosclerotic cardiovascular disease can be altered by reducing LDL-C levels (30), thereby mitigating the risk of its occurrence. In the context of breast cancer, a notable association has been established between HDL-C levels and the risk of developing breast cancer (31), with studies demonstrating a significant inverse relationship between HDL-C levels and triple-negative breast cancer (17). While elevated HDL-C levels are not a necessary factor in the onset of TNBC, they hold prognostic significance for tumor recurrence and cancer-specific mortality in TNBC patients. Consequently, monitoring and standardizing HDL levels in TNBC subtypes may be of particular importance. Further investigation into the association between blood lipids and breast cancer prognosis is warranted to enhance the targeted prevention and treatment of breast cancer recurrence or metastasis.

The rising prevalence of breast cancer on a global scale has positioned it as the second most prevalent form of cancer (32). Factors such as patient lifestyle, adjuvant therapy for breast cancer, and the significant reduction in estrogen levels due to menopause can all contribute to the development of dyslipidemia in affected individuals (Center and Society, 2022). Therefore, it is imperative to investigate the potential relationship between blood lipid levels and the prognosis of breast cancer. This systematic review and meta-analysis provides a comprehensive analysis of the existing clinical literature, highlighting the intricate relationship between blood lipid levels and breast cancer prognosis. It offers valuable insights that could enhance prognostic accuracy. However, the study is limited by several factors. Firstly, there is inconsistency in evaluating dyslipidemia across different studies, which can affect the comparability of results. Secondly, the study population demographics vary significantly, with most included studies focusing on Chinese individuals, which limits the generalizability of the findings to other racial and ethnic groups. Furthermore, the study did not perform subgroup analysis based on molecular subtypes, which could have provided more detailed insights into the relationship between blood lipids and breast cancer prognosis. Other factors such as age, breast cancer subtype, geographic location, and dietary patterns also vary across studies, potentially leading to disparate prognostic outcomes. Lastly, while the study identifies associations between blood lipid levels and breast cancer prognosis, further research, particularly through multicenter, large-sample cohort studies, is needed to more definitively establish these relationships and their clinical implications.

## 5 Conclusion

While our analysis reveals a link between reduced HDL-C levels and unfavorable survival outcomes, the statistical evidence does not support significant connections between HDL-C, TG, and LDL-C concentrations and the prognostic landscape for breast cancer patients. Further research is warranted to explore these relationships more comprehensively.

## Data Availability

The original contributions presented in the study are included in the article/**Supplementary Material.** Further inquiries can be directed to the corresponding author.
