# Supplementary material for: Impact of blood lipid levels on breast cancer prognosis: a systematic review and meta-analysis
Source: Front Oncol. 2025 Jul 1;15:1496468. doi: 10.3389/fonc.2025.1496468 (PMC12260459; doi:10.3389/fonc.2025.1496468)
Supplement: Supplementary file 1 [file DataSheet1.docx]

**Supplemental** **Box 1.**

Search strategy

| **HDL-C：**  ("Lipoproteins, HDL"[Mesh]) OR ((((((((((((((((HDL Lipoproteins[Title/Abstract]) OR (High-Density Lipoprotein[Title/Abstract])) OR (Lipoprotein, High-Density[Title/Abstract])) OR (High-Density Lipoproteins[Title/Abstract])) OR (High Density Lipoproteins[Title/Abstract])) OR (Lipoproteins, High-Density[Title/Abstract])) OR (alpha-Lipoproteins[Title/Abstract])) OR (alpha Lipoproteins[Title/Abstract])) OR (Heavy Lipoproteins[Title/Abstract])) OR (Lipoproteins, Heavy[Title/Abstract])) OR (High Density Lipoprotein[Title/Abstract])) OR (Density Lipoprotein, High[Title/Abstract])) OR (Lipoprotein, High Density[Title/Abstract])) OR (alpha-Lipoprotein[Title/Abstract])) OR (alpha Lipoprotein[Title/Abstract])) OR (alpha-1 Lipoprotein[Title/Abstract]))  AND  ("Breast Neoplasms"[Mesh]) OR (((((((((((((((((((((((((((((((((((Breast Neoplasm[Title/Abstract]) OR (Neoplasm, Breast[Title/Abstract])) OR (Breast Tumors[Title/Abstract])) OR (Breast Tumor[Title/Abstract])) OR (Tumor, Breast[Title/Abstract])) OR (Tumors, Breast[Title/Abstract])) OR (Neoplasms, Breast[Title/Abstract])) OR (Breast Cancer[Title/Abstract])) OR (Cancer, Breast[Title/Abstract])) OR (Mammary Cancer[Title/Abstract])) OR (Cancer, Mammary[Title/Abstract])) OR (Cancers, Mammary[Title/Abstract])) OR (Mammary Cancers[Title/Abstract])) OR (Malignant Neoplasm of Breast[Title/Abstract])) OR (Breast Malignant Neoplasm[Title/Abstract])) OR (Breast Malignant Neoplasms[Title/Abstract])) OR (Malignant Tumor of Breast[Title/Abstract])) OR (Breast Malignant Tumor[Title/Abstract])) OR (Breast Malignant Tumors[Title/Abstract])) OR (Cancer of Breast[Title/Abstract])) OR (Cancer of the Breast[Title/Abstract])) OR (Mammary Carcinoma, Human[Title/Abstract])) OR (Carcinoma, Human Mammary[Title/Abstract])) OR (Carcinomas, Human Mammary[Title/Abstract])) OR (Human Mammary Carcinomas[Title/Abstract])) OR (Mammary Carcinomas, Human[Title/Abstract])) OR (Human Mammary Carcinoma[Title/Abstract])) OR (Human Mammary Neoplasms[Title/Abstract])) OR (Neoplasm, Human Mammary[Title/Abstract])) OR (Neoplasms, Human Mammary[Title/Abstract])) OR (Mammary Neoplasm, Human[Title/Abstract])) OR (Breast Carcinoma[Title/Abstract])) OR (Breast Carcinomas[Title/Abstract])) OR (Carcinoma, Breast[Title/Abstract])) OR (Carcinomas, Breast[Title/Abstract])) |
| --- |
| **LDL-C：**  ("Lipoproteins, LDL"[Mesh]) OR (((((((((((((((((((((LDL Lipoproteins[Title/Abstract]) OR (beta-Lipoprotein[Title/Abstract])) OR (beta Lipoprotein[Title/Abstract])) OR (Low-Density Lipoproteins[Title/Abstract])) OR (Lipoproteins, Low-Density[Title/Abstract])) OR (Low Density Lipoproteins[Title/Abstract])) OR (beta-Lipoproteins[Title/Abstract])) OR (beta Lipoproteins[Title/Abstract])) OR (Low-Density Lipoprotein[Title/Abstract])) OR (Lipoprotein, Low-Density[Title/Abstract])) OR (Low Density Lipoprotein[Title/Abstract])) OR (LDL-2[Title/Abstract])) OR (LDL2[Title/Abstract])) OR (Low-Density Lipoprotein 2[Title/Abstract])) OR (Low Density Lipoprotein 2[Title/Abstract])) OR (LDL(2[Title/Abstract]))) OR (LDL-1[Title/Abstract])) OR (LDL1[Title/Abstract])) OR (Low-Density Lipoprotein 1[Title/Abstract])) OR (Low Density Lipoprotein 1[Title/Abstract])) OR (LDL(1[Title/Abstract])))  AND  ("Breast Neoplasms"[Mesh]) OR (((((((((((((((((((((((((((((((((((Breast Neoplasm[Title/Abstract]) OR (Neoplasm, Breast[Title/Abstract])) OR (Breast Tumors[Title/Abstract])) OR (Breast Tumor[Title/Abstract])) OR (Tumor, Breast[Title/Abstract])) OR (Tumors, Breast[Title/Abstract])) OR (Neoplasms, Breast[Title/Abstract])) OR (Breast Cancer[Title/Abstract])) OR (Cancer, Breast[Title/Abstract])) OR (Mammary Cancer[Title/Abstract])) OR (Cancer, Mammary[Title/Abstract])) OR (Cancers, Mammary[Title/Abstract])) OR (Mammary Cancers[Title/Abstract])) OR (Malignant Neoplasm of Breast[Title/Abstract])) OR (Breast Malignant Neoplasm[Title/Abstract])) OR (Breast Malignant Neoplasms[Title/Abstract])) OR (Malignant Tumor of Breast[Title/Abstract])) OR (Breast Malignant Tumor[Title/Abstract])) OR (Breast Malignant Tumors[Title/Abstract])) OR (Cancer of Breast[Title/Abstract])) OR (Cancer of the Breast[Title/Abstract])) OR (Mammary Carcinoma, Human[Title/Abstract])) OR (Carcinoma, Human Mammary[Title/Abstract])) OR (Carcinomas, Human Mammary[Title/Abstract])) OR (Human Mammary Carcinomas[Title/Abstract])) OR (Mammary Carcinomas, Human[Title/Abstract])) OR (Human Mammary Carcinoma[Title/Abstract])) OR (Human Mammary Neoplasms[Title/Abstract])) OR (Neoplasm, Human Mammary[Title/Abstract])) OR (Neoplasms, Human Mammary[Title/Abstract])) OR (Mammary Neoplasm, Human[Title/Abstract])) OR (Breast Carcinoma[Title/Abstract])) OR (Breast Carcinomas[Title/Abstract])) OR (Carcinoma, Breast[Title/Abstract])) OR (Carcinomas, Breast[Title/Abstract])) |
| **TC：**  (Epicholesterol[Title/Abstract]) OR ("Cholesterol"[Mesh])  AND  ("Breast Neoplasms"[Mesh]) OR (((((((((((((((((((((((((((((((((((Breast Neoplasm[Title/Abstract]) OR (Neoplasm, Breast[Title/Abstract])) OR (Breast Tumors[Title/Abstract])) OR (Breast Tumor[Title/Abstract])) OR (Tumor, Breast[Title/Abstract])) OR (Tumors, Breast[Title/Abstract])) OR (Neoplasms, Breast[Title/Abstract])) OR (Breast Cancer[Title/Abstract])) OR (Cancer, Breast[Title/Abstract])) OR (Mammary Cancer[Title/Abstract])) OR (Cancer, Mammary[Title/Abstract])) OR (Cancers, Mammary[Title/Abstract])) OR (Mammary Cancers[Title/Abstract])) OR (Malignant Neoplasm of Breast[Title/Abstract])) OR (Breast Malignant Neoplasm[Title/Abstract])) OR (Breast Malignant Neoplasms[Title/Abstract])) OR (Malignant Tumor of Breast[Title/Abstract])) OR (Breast Malignant Tumor[Title/Abstract])) OR (Breast Malignant Tumors[Title/Abstract])) OR (Cancer of Breast[Title/Abstract])) OR (Cancer of the Breast[Title/Abstract])) OR (Mammary Carcinoma, Human[Title/Abstract])) OR (Carcinoma, Human Mammary[Title/Abstract])) OR (Carcinomas, Human Mammary[Title/Abstract])) OR (Human Mammary Carcinomas[Title/Abstract])) OR (Mammary Carcinomas, Human[Title/Abstract])) OR (Human Mammary Carcinoma[Title/Abstract])) OR (Human Mammary Neoplasms[Title/Abstract])) OR (Neoplasm, Human Mammary[Title/Abstract])) OR (Neoplasms, Human Mammary[Title/Abstract])) OR (Mammary Neoplasm, Human[Title/Abstract])) OR (Breast Carcinoma[Title/Abstract])) OR (Breast Carcinomas[Title/Abstract])) OR (Carcinoma, Breast[Title/Abstract])) OR (Carcinomas, Breast[Title/Abstract])) |
| **TG：**  ("Triglycerides"[Mesh]) OR (((Triacylglycerols[Title/Abstract]) OR (Triacylglycerol[Title/Abstract])) OR (Triglyceride[Title/Abstract]))  AND  ("Breast Neoplasms"[Mesh]) OR (((((((((((((((((((((((((((((((((((Breast Neoplasm[Title/Abstract]) OR (Neoplasm, Breast[Title/Abstract])) OR (Breast Tumors[Title/Abstract])) OR (Breast Tumor[Title/Abstract])) OR (Tumor, Breast[Title/Abstract])) OR (Tumors, Breast[Title/Abstract])) OR (Neoplasms, Breast[Title/Abstract])) OR (Breast Cancer[Title/Abstract])) OR (Cancer, Breast[Title/Abstract])) OR (Mammary Cancer[Title/Abstract])) OR (Cancer, Mammary[Title/Abstract])) OR (Cancers, Mammary[Title/Abstract])) OR (Mammary Cancers[Title/Abstract])) OR (Malignant Neoplasm of Breast[Title/Abstract])) OR (Breast Malignant Neoplasm[Title/Abstract])) OR (Breast Malignant Neoplasms[Title/Abstract])) OR (Malignant Tumor of Breast[Title/Abstract])) OR (Breast Malignant Tumor[Title/Abstract])) OR (Breast Malignant Tumors[Title/Abstract])) OR (Cancer of Breast[Title/Abstract])) OR (Cancer of the Breast[Title/Abstract])) OR (Mammary Carcinoma, Human[Title/Abstract])) OR (Carcinoma, Human Mammary[Title/Abstract])) OR (Carcinomas, Human Mammary[Title/Abstract])) OR (Human Mammary Carcinomas[Title/Abstract])) OR (Mammary Carcinomas, Human[Title/Abstract])) OR (Human Mammary Carcinoma[Title/Abstract])) OR (Human Mammary Neoplasms[Title/Abstract])) OR (Neoplasm, Human Mammary[Title/Abstract])) OR (Neoplasms, Human Mammary[Title/Abstract])) OR (Mammary Neoplasm, Human[Title/Abstract])) OR (Breast Carcinoma[Title/Abstract])) OR (Breast Carcinomas[Title/Abstract])) OR (Carcinoma, Breast[Title/Abstract])) OR (Carcinomas, Breast[Title/Abstract])) |
